# Evaluating the feasibility of a novel device for enhanced control of chordal length assessment in mitral valve surgery: A porcine model study

**DOI:** 10.1016/j.xjtc.2026.102319

**Published:** 2026-03-19

**Authors:** Thomas Poschner, Gianluca Dimonte, Markus Mach, Andrei-Antonio Caracioni, Thomas Putz, Sahra Tasdelen, Siya Saidian, Robert E. Bartz, Jude S. Sauer, Martin Andreas

**Affiliations:** a Division of Cardiac Surgery, Medical University of Graz, Graz, Austria; b Christian Doppler Laboratory for Microinvasive Heart Surgery, Division of Cardiac Surgery, Medical University of Graz, Graz, Austria; c LSI SOLUTIONS, Victor, NY; d Division of Cardiac Surgery, University of Rochester Medical Center, Rochester, NY

**Keywords:** mitral valve repair, mitral regurgitation, chordal replacement, ePTFE, novel device, preclinical study

## Abstract

**Objective:**

Determining the appropriate expanded polytetrafluoroethylene (ePTFE) chord length remains a critical step in mitral valve repair. Freehand ePTFE suturing and premeasured loops are widely used but present technical challenges. A novel chordal holder device was developed to temporarily secure the ePTFE suture during pressurized saline infusion testing, enabling precise subsequent adjustment of chordal length. This feasibility study evaluated this technology in an ex vivo porcine heart model.

**Methods:**

In 20 ex vivo porcine hearts, Carpentier type II mitral regurgitation was induced by cutting chordae tendineae in the A2 and/or P2 segments. An LS-5 ePTFE suture was placed using the Mi-STITCH Device. The study technology temporarily held the ePTFE suture at a desired length during infusion testing. Once optimal coaptation was achieved, the chord was permanently secured with a customized Mi-KNOT titanium fastener. Times for suture placement, length adjustment, knot fixation, and total implantation were recorded.

**Results:**

A total of 37 ePTFE sutures were implanted. The chordal holder provided reliable temporary fixation and facilitated readjustment with no suture damage. Median time for valve repair was 3 minutes, 20 seconds (02:21; 5:14), with 1 to 4 ePTFE suture placements required to achieve adequate repair. Procedural times did not differ between isolated and nonisolated P2 prolapse (*P* = .8265). Final infusion testing showed no residual mitral regurgitation greater than trace in all specimens.

**Conclusions:**

Setting appropriate replacement chordal length with the evaluated technology is fast, feasible, and intuitive. The chordal holder enabled precise length adjustment and competent repairs. These encouraging results support further evaluation in clinical settings.

## Graphical Abstract

A novel device enables temporary chordal fixation and iterative pressurized saline infusion testing for precise length adjustment, resulting in efficient mitral valve repair.

**Figure:**
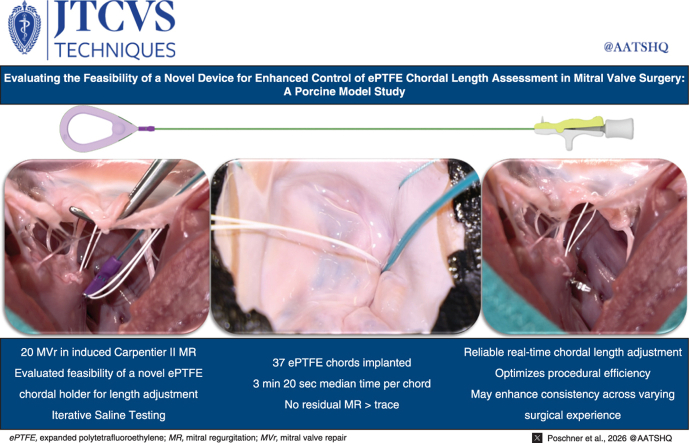

**Figure:**
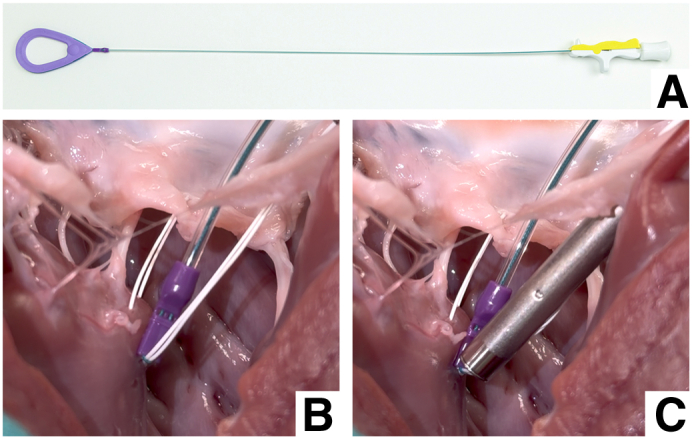
Chordal holder device (A): tip close-up during suture length assessment (B), fastening (C).

**Central Message**

This technology holds the chordal suture during infusion testing to enable evaluation of chordal length for optimized mitral valve leaflet coaptation before the suture is permanently secured.

**Perspective**

Determining the correct chordal length remains one of the most critical steps in mitral valve repair (MVr). In this evaluation, a novel technology to maintain chordal length during pressurized saline infusion testing facilitated precise replacement chord length adjustment before securing the suture. Future investigations of this encouraging technology may further improve reliability in MVr.

Surgical mitral valve repair (MVr) remains the preferred therapy for degenerative mitral regurgitation (MR) because of its durability and proven survival benefit.^1^^,^^2^ Although there is an ongoing debate regarding “resect” versus “respect” approaches, these concepts should be viewed as complementary options toward a personalized, pathology-driven repair strategy rather than an either-or decision.^3^, ^4^, ^5^ The “respect” approach, which relies on expanded polytetrafluoroethylene (ePTFE) artificial chordae, can preserve leaflet tissue and motion while enabling broad coaptation and generally a larger orifice area.^3^ However, correct length assessment of slippery ePTFE chordae, especially in the setting of minimally invasive cardiac surgery, remains challenging.^6^ As microinvasive heart surgery approaches continue to develop, there is an unmet clinical need for additional options to overcome the perceived technical difficulty of existing techniques.

Premeasured ePTFE loops are increasingly being used, purportedly to simplify implantation.^7^^,^^8^ However, ensuring appropriate attachment to the papillary muscle and leaflet remains somewhat subjective. This loop technique requires keeping suture knots near the coaptation zone. In addition, pressurized infusion testing by injection of saline into the arrested left ventricle typically necessitates temporary fixation of the loops at their attachment sites. Alternative techniques have been described to provide frictional stability during pressurized saline testing.^9^, ^10^, ^11^ However, the need for repeated loosening and retightening is technically challenging and time-consuming, particularly in minimally invasive procedures. Consequently, the complexity of these adjustments may force the surgeons to accept a potentially suboptimal repair in favor of efficiency.

New technologies have recently been introduced to address this limitation by facilitating individualized, reproducible chordae placement.^12^^,^^13^ One such system, the Mi-CHORD platform (LSI SOLUTIONS) enables rapid and secure ePTFE placement and fastening. Early clinical experience demonstrated promising feasibility and sufficient repair.^12^ Still, final chordal length determination remains dependent on the surgeon's judgment and experience. The adjunctive device presented in this study is intended to facilitate reliable chordal length adjustment by minimizing subjective length assessment during saline testing.

To further enhance the reproducibility while preserving flexibility, a chordal-holding device was developed to temporarily secure ePTFE suture length during intraoperative saline testing. This facilitates incremental adjustments of chordal length during repeated saline testing, allowing the surgeon to reset the chordal length toward optimization of coaptation before final fixation. This preclinical study evaluated the feasibility of device-assisted, real-time chordal length adjustment in an ex vivo porcine model simulating degenerative MR.

## Methods

Porcine hearts were used without chemical preservation. Each specimen was positioned in a custom holder that maintained ventricular orientation and provided direct access to the mitral valve after partial excision of the left atrium. Valve competence was assessed by retrograde saline testing through a 16-Fr catheter advanced across the aortic valve into the left ventricle and secured with a running suture. MR was induced by cutting several native chordae tendineae of either the posterior or anterior leaflet to create a degenerative mitral valve pathology (Carpentier Class II).^14^

Artificial chordae (ePTFE sutures) were implanted using the Mi-STITCH device (LSI SOLUTIONS). After the ePTFE suture was placed near the edge of the prolapsing leaflet tissue and through the corresponding papillary muscle, its free suture ends were passed through the chordal holder to temporarily hold the replacement chordal suture at a chosen length. This device consists of a single green size 0 polyester suture loop that runs through separate channels in the dual-lumen tip at the patientʼs end, a suture tube, and a manually opened and closed locking mechanism at the surgeonʼs end. The technology is intended to be an easily used, atraumatic suture holder, with a very low-profile tube to enable unimpaired pressurized saline infusion testing. The device allows the surgeon to set and hold the chordal suture at a certain length and potentially adjust then resecure the suture, based on results of the infusion testing. This process can be repeated as desired to achieve optimized leaflet coaptation. After achieving satisfactory infusion testing, the chordal holder remains in place to maintain the desired ePTFE suture length as the suture is secured with a Mi-KNOT titanium fastener. The chordal holder is subsequently removed and a final infusion test is performed to confirm optimal leaflet coaptation and determine whether additional ePFTE chordae are needed (Video 1). Once the repair was deemed acceptable, pressurized saline infusion testing was performed in all specimens. Intraventricular pressure was measured using a manometer in the final 10 specimens, with pressure consistently exceeding 180 mm Hg (see Table E1). The same protocol was applied for the preceding 10 hearts; however, intraventricular pressure was not measured in those experiments. Potential residual MR was assessed visually. Complete absence of regurgitation was graded as none and minimal leakage as trace.

Times for the procedure were recorded as the sum of suture placement, length adjustment with iterative saline testing, and knot fixation. The head of the department (surgeon A) performed an MVr in 5 of the heart specimens; an attending surgeon (surgeon B) performed another 5 MVr; and a fourth-year cardiac surgery resident (surgeon C) performed MVr on the remaining 10 heart specimens.

Institutional approval was not applicable for this study. Only hearts from the local slaughterhouse were used. No human subjects were involved. Therefore, informed consent was not applicable.

Statistical comparisons between isolated P2 versus nonisolated P2 prolapse, as well as with Mi-STITCH first-in-human data, were performed using the Mann-Whitney *U* test. A comparison between the surgeons was performed using the Kruskal-Wallis test. Median values and interquartile ranges are reported.

## Results

Mitral valve regurgitation was successfully induced in all 20 porcine hearts (399 ± 28 g; range 302-461 g) by cutting native chordae: The P2 segment was targeted in 15 specimens; the A2 in 4 specimens; and combined A2/P2 segments in 1 specimens. An MVr procedure incorporating replacement ePTFE chordae was then completed in each heart. Three surgeons completed these procedures. Surgeon A performed 5 procedures with 14 total stitches (median time 2 minutes, 55 seconds) placed; surgeon A removed 2 chordae in specimen 2 to improve valve competence, leaving a total of 12 replacement chordae in place (86%). Surgeon B performed 5 procedures with a total of 5 stitches (median time 5 minutes, 14 seconds) and all were left in place (100%). Surgeon C performed the other 10 procedures, with a total of 18 stitches placed (median time 3 minutes, 38 seconds); surgeon C removed a total of 6 chordae (of those, 1 was removed because of interference with the test setup and 2 were removed to reduce observed trace residual MR to none), leaving a total of 12 replacement chordae in place (67%). Typically, each isolated P2 repair was corrected with 1 or 2 ePTFE chordae, whereas nonisolated P2 repair required up to 4 chordae. The chordal holder was used for intraoperative adjustment in all chordae except one (immediate determination of the correct length) and functioned as intended, providing secure temporary fixation and facilitating rapid readjustment without suture damage or entanglement. Table 1 summarizes the procedural details and times.

**Table 1:** Detailed case-by-case report of results

Specimen | surgeon ID	Procedure notes	Number of replacement chordae	Suturing time	Length assessment time	Knotting time	Total time
1 | A	Prolapse P2 Annuloplasty? No Residual MR? None	1	1:14	1:58	0:31	3:43
2 | A	Prolapse P2 and A2	1 (X)	0:38	1:57	0:28	3:03
	Annuloplasty? Yes	2	0:51	0:49	0:23	2:03
	Residual MR? None	3	0:55	1:20	0:16	2:31
		4	2:16	5:09	0:14	7:39
		5 (X)	3:27	0:58	0:06	4:31
		6∗	0:42	–	–	5:04
3 | C	Prolapse P2 Annuloplasty? No Residual MR? None	1	0:47	3:20	0:12	4:19
4 | C	Prolapse A2	1 (X)	1:09	1:49	0:22	3:20
	Annuloplasty? No	2	1:23	2:10	0:59	4:32
	Residual MR? None					
5 | C	Prolapse P2 Annuloplasty? No Residual MR? None	1	0:25	1:04	0:07	1:36
6 | C	Prolapse P2	1 (X)	0:29	5:53	0:17	6:39
	Annuloplasty? No	2 (X)	1:35	14:05	0:16	15:56
	Residual MR? Trace	3	0:36	0:42	0:11	1:29
		4	0:59	2:45	0:12	3:56
7 | C	Prolapse P2 Annuloplasty? Yes Residual MR? None	1	1:04	5:18	0:10	6:32
8 | C	Prolapse P2 Annuloplasty? No Residual MR? None	1	0:29	1:37	0:15	2:21
9 | C	Prolapse P2 Annuloplasty? No Residual MR? None	1	0:41	2:16	0:18	3:15
10 | C	Prolapse P2	1 (X)	0:42	1:01	0:16	1:59
	Annuloplasty? No	2 (X)	0:44	6:30	0:14	7:28
	Residual MR? None	3	1:13	4:55	0:18	6:26
		4	1:07	6:40	0:14	8:01
11 | A	Prolapse P2 Annuloplasty? No Residual MR None	1	0:54	0:48	0:18	2:00
12 | A	Prolapse A2 Annuloplasty? No Residual MR? Trace	1	0:46	0:55	0:37	2:18
		2	0:49	1:53 °	0:06	2:48
		3	0:43	0:48	0:16	1:47
		4	0:49	2:23	0:06	3:18
13 | A	Prolapse P2 Annuloplasty? No Residual MR? None	1	1:20	2:08 °	0:19	3:47
		2	0:42	0:50	0:17	1:49
14 | B	Prolapse P2 Annuloplasty? No Residual MR? Trace†	1	0:32	2:08 °	0:15	2:55
15 | B	Prolapse P2 Annuloplasty? No Residual MR? Trace†	1	0:27	6:51 ° °	0:12	7:30
16 | C	Prolapse P2 Annuloplasty? No Residual MR? Trace†	1 (X)	0:33	1:20	0:16	2:09
		2	0:28	2:01	0:15	2:44
17 | C	Prolapse P2 Annuloplasty? No Residual MR? None	1	0:50	1:35 °	0:16	2:41
18 | B	Prolapse P2 Annuloplasty? No Residual MR? None	1	1:02	4:19 °	0:18	5:39
19 | B	Prolapse A2 Annuloplasty? No Residual MR? None	1	1:07	3:56	0:11	5:14
20 | B	Prolapse A2 Annuloplasty? No Residual MR? Trace†	1	0:47	2:43	0:13	3:43

Stitches removed are marked with an (X) next to the number. The stitch marked with an “∗” was performed without the chordal holder and therefore not included into the total time. The number of readjustments is available only from case 11 onward; the number of readjustments is indicated by number of dots (°) next to the time. In specimens 7 and 8, an additional cleft closure was necessary. *MR*, Mitral regurgitation.

† Residual regurgitation due to pre-existing cleft at P2/P3 or P1/P2, respectively. Times are shown in minutes:seconds.

Median time for valve repair was 3 minutes, 20 seconds (02:21; 5:14). Total time for MVr in isolated P2 or nonisolated P2 prolapse did not differ significantly (*P* = .8265). The median suture placement time per chord (from chord insertion to ready-to-adjust) was 49 seconds (41; 67). Length adjustment times varied, with a median of 125 seconds (76; 242) per chord, and did not differ between isolated P2 or nonisolated P2 prolapse (*P* = .1876). Knot fixation took a median of 16 seconds (12; 18). There was no statistically significant difference in time for valve repair between surgeons for either isolated P2 or nonisolated P2 prolapse (*P* = .3462 and *P* = .1954, respectively). Comparing these results with our first-in-human trial using Mi-STITCH without the new technology, we found no significant difference in the total implantation time per chord (3 minutes, 26 seconds [2:21; 5:14] vs clinical study: 3 minutes, 31 seconds [2:40; 5:29]; *P* = .4525). However, using the chordal holder resulted in a 37% shorter time for the first stitch placement (3 minutes, 15 seconds [2:21; 4:19] vs 5 minutes, 8 seconds [2:26; 6:05]; *P* = .3062), though this difference did not reach statistical significance (*P* = .2351).

Annuloplasty with a Memo 4D ring (CORCYM S.R.L.) was performed in 3 cases for illustrative purposes only. Final saline testing was satisfactory, with 70% (n = 14) showing no residual mitral regurgitation and 6 exhibiting trace residual regurgitation. Residual regurgitation was not directly associated with the induced prolapse but was rather attributed to pre-existing clefts in 4 specimens, which either were not further addressed surgically or failed to improve despite attempted cleft closure.

## Discussion

In this preclinical study, the use of a novel technology to facilitate setting ePTFE chordal length was demonstrated to be feasible, achieving excellent results without adding substantial procedural time. This technology could be readily integrated into the MVr procedure workflow without adding procedural time in this preclinical study.

Determining the correct replacement chord length is one of the most critical steps in MVr surgery. Freehand ePTFE suturing and knot placement can be challenging and may compromise valve competence. The preameasured loop technique can enable reproducible results but lacks the flexibility of modifying length after pressurized saline infusion testing.^8^ If the repair is not satisfactory, loop replacement is cumbersome and time-consuming and can eventually lead to the acceptance of a suboptimal result or potential valve replacement.

Pressurized saline infusion testing has long been used to assess replacement chordae length. Several different techniques have been proposed, with none achieving widespread clinical adoption, mostly because they rely on improvised tools and lack reliable suture control.^11^^,^^15^, ^16^, ^17^ The evaluated chordal holder, in contrast, provides a customized solution for this challenge by stabilizing the chordal length during infusion testing while still readily allowing subsequent readjustment. Length can be refined incrementally after simulated physiologic assessment. Once suitable coaptation is demonstrated, the chordal holder maintains that length during suture securing with a customized titanium fastener, ensuring that the ePTFE chord is fixed precisely at the desired length.

The number of stitches removed ranged from 0 to 33%, depending on the surgeon. Setup interferences and attempts to eliminate trace residual regurgitation led to the removal of 3 stitches in total. Thus, only 17% of the chordae were removed and replaced as a result of the initial unsatisfactory repair result, consistent with other surgeons. Importantly, suture removal was not predefined as a negative end point, and revisions were performed liberally at the surgeons’ discretion.

The added flexibility with the chordal holder did not increase the time for valve repair compared with using the automated system alone. Time for placing and securing the first chord was actually 37% faster using the chordal holder, although not statistically significant; however, one should consider that the comparison cohort came from a clinical setting, with one third of cases performed through minimally invasive access. The time-neutral profile of this novel device is of utmost importance, especially in minimally invasive mitral surgery, during which surgeons must balance precision with efficiency. The simple mechanism of the study device proved intuitive, with a minimal learning curve. Moreover, the technology is not limited to automated devices and can be used during testing of freehand ePTFE sutures as well, reducing the risk of unintentional length changes before fixation and restoring the surgeonʼs ability to individualize the repair.

There were no statistically significant differences in total repair time between surgeon A (experienced surgeon) and either surgeon B (attending surgeon without previous exposure to the system) or surgeon C (fourth-year resident). Although numerical differences were observed, they did not reach statistical significance, underscoring the system's intuitive design. This is further supported by excellent results from an in-house usability assessment, with composite scores of 91.8 of 100 for the automated suturing device, the chordal holder, and the automated knotting device. A rapid improvement from 83 to 97 points in the last assessment demonstrates the technologyʼs short learning curve. Of note: The usability assessment was only completed by surgeon B, because the other surgeons had substantial previous exposure to the system.

Another important finding that may have significant clinical implications is the excellent results achieved with reasonable surgical times also for the inexperienced surgeons, suggesting that the implementation of the chordal holder may flatten the learning curve by turning length determination into a structured, test-based process rather than an intuitive skill slowly acquired over time. By enabling iterative refinement without prolonging the procedure, this device may support a more consistent and sophisticated repair potentially reducing early failure rates.

### Limitations

This study was performed in an ex vivo, nonbeating-heart model using static saline testing. Although this approach is common for early feasibility work, it cannot replicate physiologic loading conditions or dynamic leaflet motion. Although pressurized saline infusion testing is widely used intraoperatively, it does not reflect the hemodynamic complexity of a beating heart and may artificially flatten the mitral annulus or overstretch the leaflets.^18^ Only acute performance was evaluated; therefore, long-term suture durability after multiple length adjustments was not assessed. Moreover, the model mostly involved isolated prolapse rather than more complex lesions, such as multisegmental degeneration, annular calcification, or restricted leaflets, so only a smaller portion of clinical scenarios was evaluated. Future studies in animals and subsequent clinical trials will be essential to evaluate device performance under physiological conditions.

## Conclusions

The evaluated novel chordal holder technology was demonstrated to be effective and easy to use for maintaining suture length to facilitate pressurized saline infusion testing. This technology allowed the surgeons to optimize mitral leaflet coaptation on the basis of the reliable results of simulated physiologic testing and secure ePTFE chordae at their desired length. These results support further evaluation of using this technology to facilitate chordal repair in animal models and subsequent clinical trials to improve the reliability of MVr with chordal replacement.

### Declaration of Generative AI and AI-Assisted Technologies in the Writing Process

During the preparation of this work the authors used Grammarly, Inc, for linguistic enhancement in preparation of the manuscript. After using this tool, the authors reviewed and edited the content as needed and take full responsibility for the content of the publication.

## Conflict of Interest Statement

T.P. received travel grants from Abiomed, Biotronik, and LSI SOLUTIONS. G.D. and S.T. were recipients of travel grants from LSI SOLUTIONS. M.M. received research grants from Edwards Lifesciences Corp, JenaValve Technology, Inc, Symetis SA, Medtronic plc, Abbott Laboratories, Novartis AG, Boston Scientific Corporation, and LivaNova PLC. S.S., R.E.B., and J.S.S. are full-time employees of LSI SOLUTIONS. J.S.S. is founder and owner of LSI SOLUTIONS and inventor of the subject technology. M.A. is a proctor/consultant/speaker (Abbott, Edwards, Medtronic, Boston Scientific Corporation, B. Braun, Zoll) and received institutional research grants (Abbott, Edwards, Medtronic, LSI SOLUTIONS). All other authors have nothing to declare.

The *Journal* policy requires editors and reviewers to disclose conflicts of interest and to decline handling or reviewing manuscripts for which they may have a conflict of interest. The editors and reviewers of this article have no conflicts of interest.

## Figures and Tables

**Table E1:** Measured maximal intraventricular pressures (last 10 cases studied)

Case	Peak pressure, mm Hg
11	200
12	220
13	220
14	200
15	180
16	220
17	250
18	220
19	200
20	220

## Supplementary Data

Video 1 Representative video of an open-heart view illustrating the step-by-step induction of Carpentier type II mitral regurgitation, automated ePTFE chordae implantation and length assessment with the chordal holder, and suture fixation with a customized titanium fastener. Then the actual test scenario is shown, demonstrating final valve evaluation with trace residual MR. Video available at: https://www.jtcvs.org/article/S2666-2507(26)00126-4/fulltext.
