# Supplementary figures and images for: Evaluating the feasibility of a novel device for enhanced control of chordal length assessment in mitral valve surgery: A porcine model study
Source: JTCVS Tech. 2026 Mar 19;37:102319. doi: 10.1016/j.xjtc.2026.102319 (PMC13261156; doi:10.1016/j.xjtc.2026.102319)

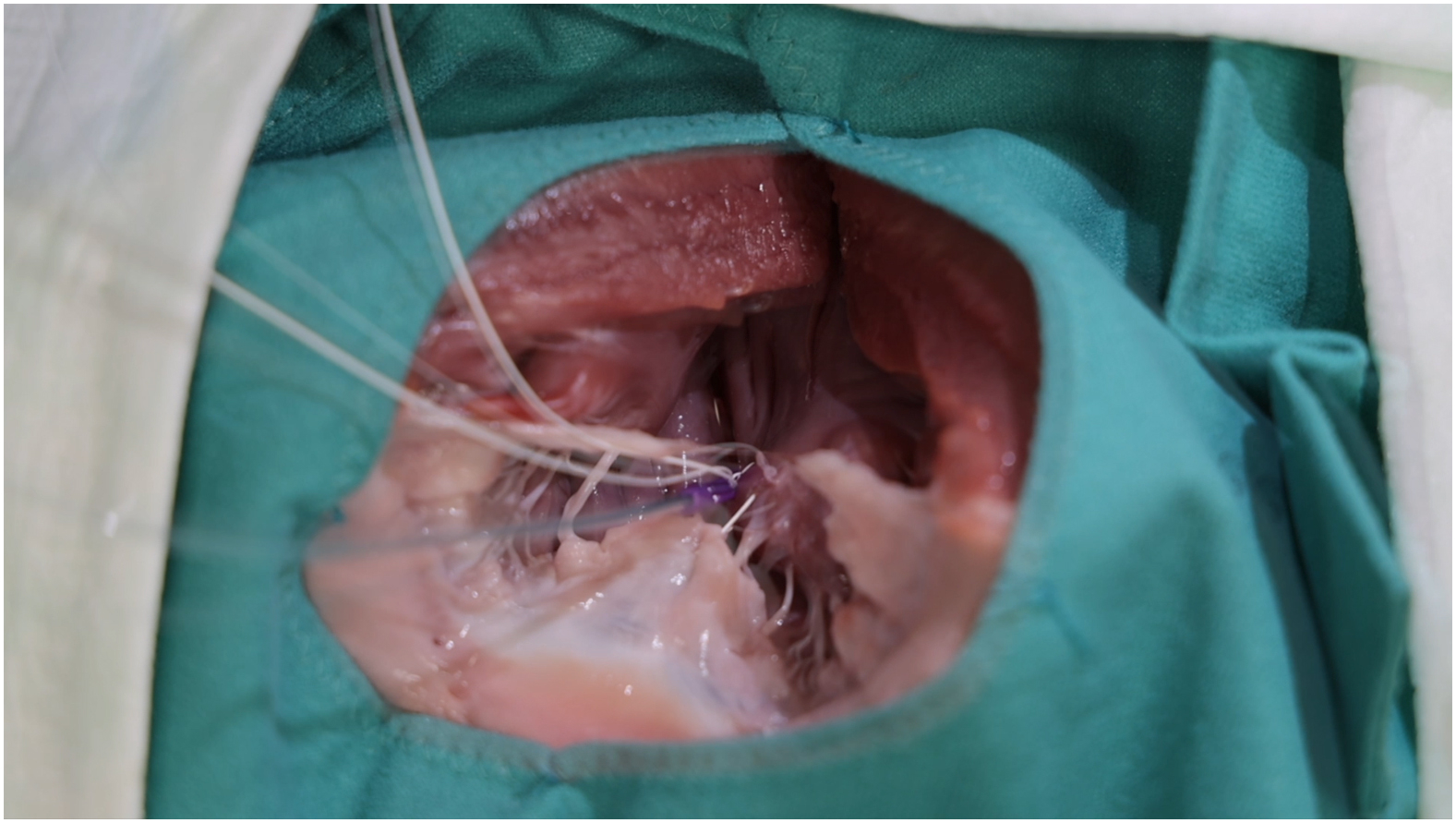

Supplement: Video 1 — Representative video of an open-heart view illustrating the step-by-step induction of Carpentier type II mitral regurgitation, automated ePTFE chordae implantation and length assessment with the chordal holder, and suture fixation with a customized titanium fastener. Then the actual test scenario is shown, demonstrating final valve evaluation with trace residual MR. Video available at: https://www.jtcvs.org/article/S2666-2507(26)00126-4/fulltext. [file fx2.jpg]
